# High-Degree Atrioventricular Block in a Patient With Asymptomatic COVID-19 Infection: A Case Report

**DOI:** 10.7759/cureus.24397

**Published:** 2022-04-22

**Authors:** Nodari Maisuradze, Ola Rehaw, David Maglakelidze, Adam S Budzikowski, Ahmad Jallad

**Affiliations:** 1 Internal Medicine, State University of New York Downstate Medical Center, Brooklyn, USA; 2 Internal Medicine, University of Alabama at Birmingham, Birmingham, USA; 3 Internal Medicine, Coney Island Hospital, Brooklyn, USA; 4 Cardiology, State University of New York Downstate Medical Center, Brooklyn, USA

**Keywords:** high-degree av block, covid-19, ventricular paced rhythm, av block, cardiac pacemaker

## Abstract

The first cases of COVID-19 infection were reported as pneumonia of unknown cause in China in December 2019. While respiratory complications remain the hallmark of the disease, multisystem involvement has been well documented. Cardiovascular involvement with potentially lethal myocarditis has been extensively reported in the literature. Reports of conduction system disturbances are much rarer, especially in patients without other signs of cardiac involvement. We present a case of an 88-year-old male with no prior cardiac history who presented to the hospital with obstipation. He was diagnosed with a small bowel obstruction and underwent a lysis of adhesions. During the hospitalization, he developed intermittent bradycardia with a high-degree atrioventricular (AV) block. A decision was made to implant a permanent pacemaker. During a pre-procedure COVID-19 screen, he was found to be positive for the presence of SARS-CoV-2 RNA. He had no signs of myocardial injury, a transthoracic echocardiogram showed no abnormalities, and he remained free of any respiratory symptoms. While the involvement of the cardiac conduction system has been documented in patients with symptomatic COVID-19 infection, our patient only exhibited conduction abnormalities and remained free of other COVID-19 symptoms. The sole involvement of the conduction system by COVID-19 is rare, especially in patients with otherwise asymptomatic infections. There is no long-term data to suggest whether such conduction abnormalities are temporary or permanent. As such, patients might benefit from the implantation of a permanent pacemaker.

## Introduction

The first cases of COVID-19 infection were reported as pneumonia of unknown cause in Wuhan City, China, in December 2019 [1]. While respiratory complications remain the hallmark of COVID-19, cardiovascular system involvement has been well documented [2]. Early reports confirmed a high prevalence of myocardial damage and its association with increased mortality [3]. Dagher et al. described a case series of four patients infected with COVID-19 developing a high-degree atrioventricular (AV) block [4]. All four patients described in the case series mentioned had severe respiratory complications requiring supplemental oxygen [4]. There is a limited number of case reports of asymptomatic patients with COVID-19 without laboratory or imaging evidence of cardiac involvement developing high-degree AV conduction disturbances. We present a case report of a patient with an asymptomatic COVID-19 infection and a high-degree AV block requiring permanent pacemaker implantation.

## Case presentation

An 88-year-old male with a past medical history of hypertension, diabetes mellitus, and hyperlipidemia presented to the hospital with a five-day history of obstipation.

**Figure 1:**
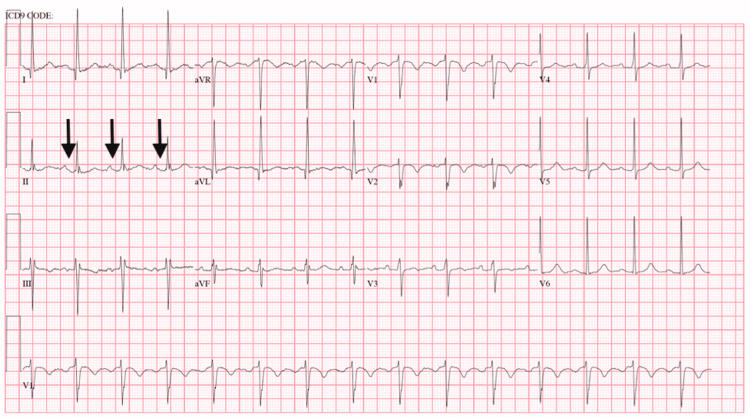
Baseline ECG Black arrows pointing at the normal PR intervals

The patient was ultimately diagnosed with a small bowel obstruction due to intestinal adhesions. As a part of a routine preadmission assessment, the patient was tested negative for the presence of SARS-CoV-2 RNA using a nasopharyngeal swab. His admission electrocardiogram (ECG) revealed a normal sinus rhythm with normal PR (188 ms) and QRS (86 ms) intervals without acute ST-T abnormalities (Figure 1). Other laboratory results obtained on admission revealed hypokalemia of 3 mEq/L and a creatinine level of 1.7 mg/dL elevated above his normal baseline consistent with poor oral intake. The patient was placed in a surgical unit on telemetry monitoring and later underwent an uneventful exploratory laparotomy for lysis of adhesions. The patient had an uneventful initial postoperative course and regained a normal bowel function.

**Figure 2:**
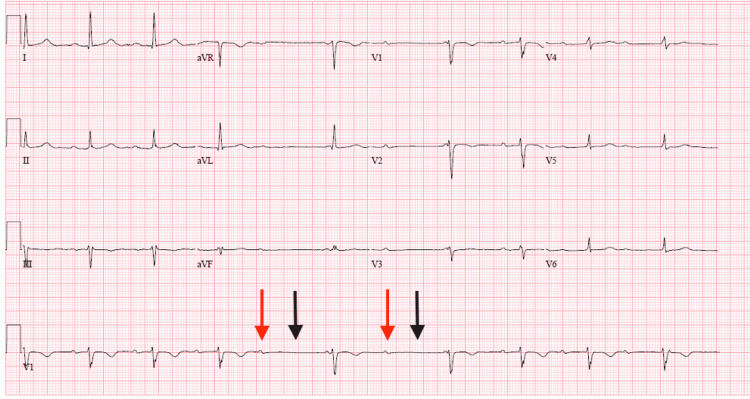
ECG with intermittent high-degree AV block Red arrow pointing at the p wave, black arrow pointing at a "dropped" QRS, followed by a junctional QRS

On postoperative day 6, the patient was noted to have recurrent episodes of asymptomatic bradycardia on telemetry. At the time, the rhythm was consistent with intermittent 2:1 AV block with a heart rate of 30s. A 12-lead electrocardiogram obtained during an episode of bradycardia revealed a high-degree AV block with no acute ST-T changes (Figure 2). Given the high-degree AV block, a decision was made to implant a permanent pacemaker. As a part of routine pre-procedure testing, the patient was found to have a positive SARS-CoV-2 test. The patient was free of any respiratory symptoms and did not suffer from hypoxia.

The patient did not have episodes of bradycardia or AV block during the first six days of hospitalization when he was presumed to be COVID-19-negative nor did he have a history of syncope or dizziness. The patient had a negative troponin I level on admission, as well as eight days later. He had a recent transthoracic echocardiogram that revealed a normal left ventricular ejection fraction (LVEF) and no regional wall motion abnormalities to suggest ischemic heart disease. The patient did not receive any medications with known negative chronotropic effects.

**Figure 3:**
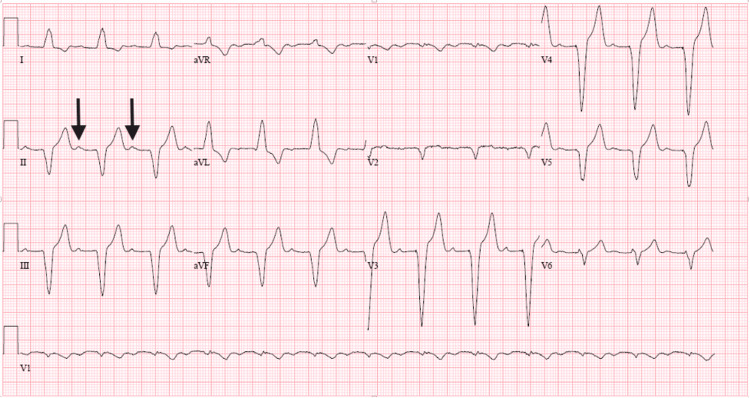
Twelve-lead ECG six months after pacemaker implantation with atrial-sensed, ventricular-paced rhythm Black arrows pointing at a p wave, followed by a prolonged PR interval, followed by a paced QRS

At his six-month follow-up visit after the pacemaker implantation, the patient reported no symptoms of dizziness or syncope. His 12-lead ECG demonstrated an atrial-sensed, ventricular-paced rhythm with a PR interval of 274 ms (Figure 3). His device interrogation revealed that the patient was ventricularly paced 59% of the time.

## Discussion

We present a case of a patient with COVID-19 who developed recurrent paroxysmal high-degree AV block necessitating implantation of a permanent pacemaker. The cardiovascular effects of SARS-CoV-2 have been well documented in critically ill patients. Our patient did not exhibit any respiratory symptoms attributable to COVID-19. He had two troponin I levels eight days apart within the normal reference range. His transthoracic echocardiogram was unrevealing with a normal LVEF and no regional wall motion abnormalities. The telemetry strip and an ECG demonstrated a 2:1 AV block without an associated decrease in sinus node activity, suggesting an AV nodal rather than a vagus nerve involvement. In our patient, whether the AV block was permanent or temporary was uncertain, driving the decision to implant a permanent pacemaker. The pacemaker was interrogated six months later, demonstrating the patient to be ventricularly paced 59% of the time despite the paced and sensed atrioventricular delay being set to >250 ms.

Given that the patient remained asymptomatic during the episodes of bradycardia, we cannot exclude the possibility of latent AV block before the discovery on telemetry. However, given no signs of bradycardia during the first six days of hospitalization, followed by frequent episodes of high-degree AV block, conduction disturbance can be attributed to the newly acquired SARS-CoV-2 infection.

## Conclusions

At present, data regarding the involvement of the cardiac conduction system by SARS-CoV-2 is limited, especially in patients with asymptomatic infections. It is unclear whether these disturbances are permanent or temporary. As such, implantation of permanent pacemakers may be warranted as demonstrated by the prolonged pacing requirement in our patient.
